# Combinations of genetic data in a study of oral cancer

**DOI:** 10.18632/genesandcancer.79

**Published:** 2015-09

**Authors:** Erling Mellerup, Gert Lykke Moeller, Pinaki Mondal, Susanta Roychoudhury

**Affiliations:** ^1^ Laboratory of Neuropsychiatry, Department of Neuroscience and Pharmacology, Faculty of Health, University of Copenhagen, Denmark; ^2^ Genokey ApS, ScionDTU, Technical University of Denmark, Hoersholm, Denmark; ^3^ National Brain Research Centre, Gurgaon, India; ^4^ Cancer Biology and Inflammatory Disorder Division, CSIR-Indian Institute of Chemical Biology, Kolkata, India

**Keywords:** combinations, genetic data, oral cancer, leukoplakia, genetic subgroups

## Abstract

In the single locus strategy a number of genetic variants are analyzed, in order to find variants that are distributed significantly different between controls and patients. A supplementary strategy is to analyze combinations of genetic variants. A combination that is the genetic basis for a polygenic disorder will not occur in in control persons genetically unrelated to patients, so the strategy is to analyze combinations of genetic variants present exclusively in patients. In a previous study of oral cancer and leukoplakia 325 SNPs were analyzed. This study has been supplemented with an analysis of combinations of two SNP genotypes from among the 325 SNPs. Two clusters of combinations containing 95 patient specific combinations were significantly associated with oral cancer or leukoplakia. Of 373 patients with oral cancer 205 patients had a number of these 95 combinations in their genome, whereas none of 535 control persons had any of these combinations in their genome.

## INTRODUCTION

A specific combination of genetic changes is the genetic basis for a polygenic disorder; this combination can be found in patients, but not in control subjects genetically unrelated to patients. If the disorder shows genetic heterogeneity several combinations of genetic variants may be basis for the disorder. Molecular genetic studies of many diseases have identified a number of individual genetic variants contributing to the risk of disease. The effect size for most of these variants is small, which has led to the concept of missing heritability [1]. Combinations of genetic variants may contribute to the heritability; but, studies of combinations are rare, due to the computational and statistical challenges created by the large number of possible combinations, even with moderate numbers of genetic variants [2]. This problem can be addressed by restricting the analysis to a small number of genetic variants [3,4], by development of fast data mining methods [5,6,7], and by using specialized hardware, as multiple graphical processing units, to increase scanning speed [8,9]. Using such types of methods combinations of genetic variants have been analyzed in studies of esophageal cancer [10], bipolar disorder [11], neuroblastoma [12], and breast cancer [13]. In the present study combinations of two single nucleotide polymorphism (SNP) genotypes taken from among 325 SNPs were analyzed, the SNPs were from a previous study of oral cancer and leukoplakia [14].

## RESULTS

**Figure 1:**
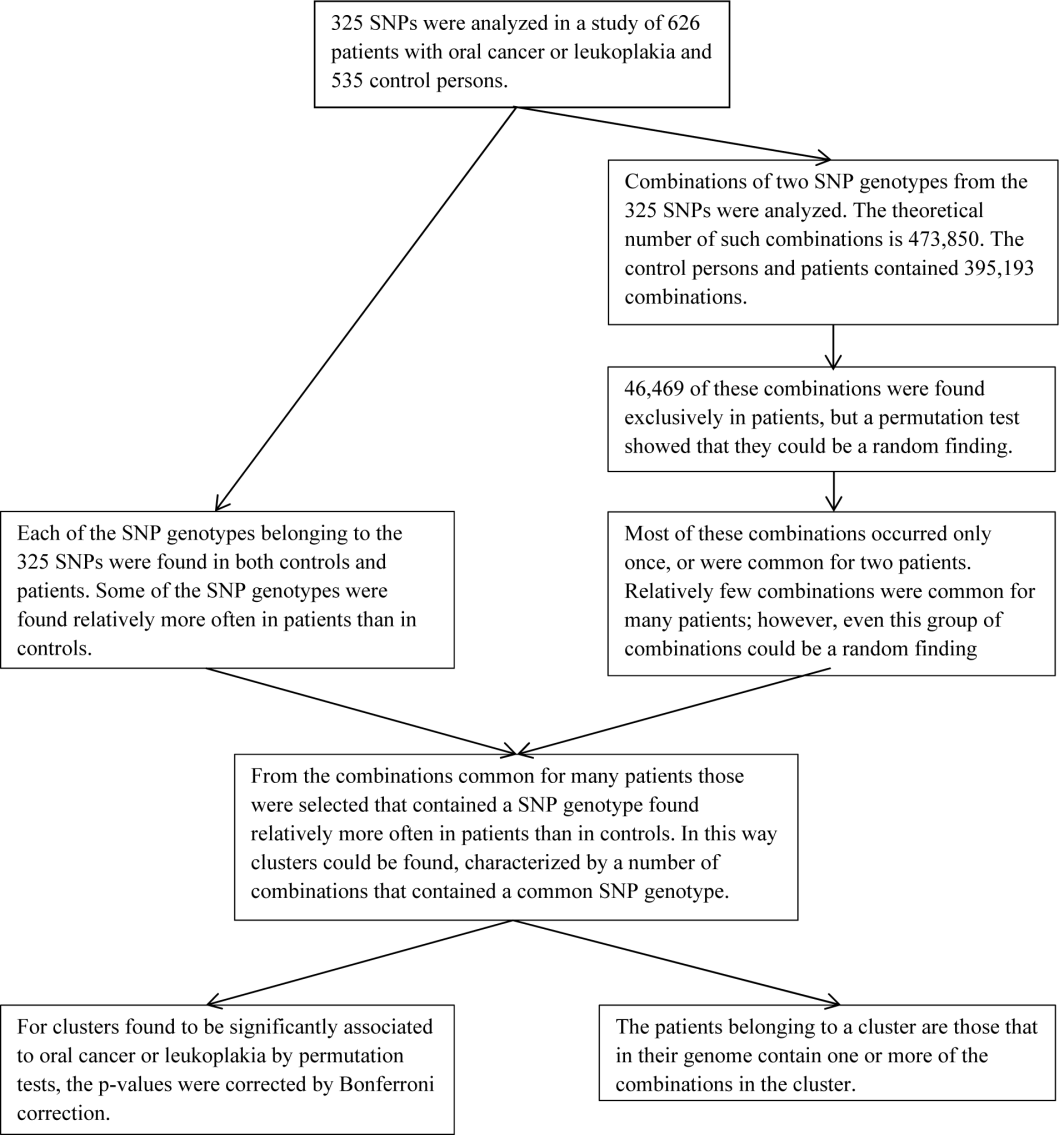
Diagram showing the steps leading from 325 SNPs to clusters of combinations of SNP genotypes significantly associated to oral cancer or leukoplakia

In a previous study of 373 patients with oral cancer, 253 patients with leukoplakia, and 535 controls, 325 SNPs from 11 genes involved in DNA repair were analyzed, one SNP genotype was found to be significantly associated with oral cancer and two SNP genotypes were significantly associated with leukoplakia (Figure 1) [14]. This study has been supplemented with an analysis of combinations of the SNP genotypes. The theoretical number of combinations of two SNP genotypes taken from 325 SNPs is (325!/(325 - 2)!2!) × 3^2^ = 473,850. In the participants 395,193 of these combinations were found, of which 328,238 were common for controls and patients, 20,486 were in controls only, and 46,469 were found exclusively in patients. Among these patient specific combinations two clusters of combinations were found to be significantly associated to oral cancer and leukoplakia (permutation test, p<0.001). Cluster 1 comprised 52 combinations which contained 32 SNP genotypes; cluster 2 comprised 43 combinations which contained 44 SNP genotypes. The patients in the clusters were those who had one or more of these combinations in their genome. Cluster 1 contained 167 oral cancer patients and 24 leukoplakia patients; cluster 2 contained 38 oral cancer patients and 16 leukoplakia patients. The first five combinations in the two clusters are shown in Table 1 and Table 2. All of the combinations are shown in Supporting S1 and S2 Tables.

**Table 1:** The combinations of two SNP genotypes in Cluster 1

SNP0^genotype^	SNP1^genotype^	Patients
rs207923^1^	rs228589^1^	51 54 59 61 68 69 75 77 78 79 82 84 85 86 87 90 91 94 95 96 98 101 102 103 104 105 107 108 109 111 112 113 114 115 117 118 121 122 124 125 126 127 129 132 133 134 137 138 141 142 143 145 146 200 216 290 332
rs8178176^1^	rs13447682^1^	63 73 77 81 87 93 100 102 106 108 109 110 112 114 115 117 119 122 123 124 128 133 135 139 145 146 180 181 182 183 199 206 208 216 223 225 226 233 236 242 244 245 246
rs8178176^1^	rs8178107^1^	55 63 65 73 74 81 87 100 106 108 109 110 112 114 115 117 119 124 135 139 145 146 180 208 215 225 226 236 245 246
rs13447682^1^	rs228589^1^	55 63 65 73 74 81 87 100 106 108 109 110 112 114 115 117 119 124 135 139 145 146 180 208 215 225 226 236 245 246
rs3797896^1^	rs2735384^1^	139 155 166 173 174 180 191 200 216 217 224 226 227 236 237 246 247 249 415 462 479 481 594 620 623 624 627 631

Only the first 5 combinations are shown. All 52 combinations in Cluster 1 are shown in supporting S1 Table. ^0,1,2^ after rs number shows genotype (0 homozygous for the major allele, 1 heterozygous, 2 homozygous for the minor allele). Patient number 0-378 indicates oral cancer, patient number 379-631 indicates leuokoplakia.

**Table 2:** The combinations of two SNP genotypes in Cluster 2

SNP0^genotype^	SNP1^genotype^	Patients
rs16901941^0^	rs132774^2^	17 45 71 83 157 169 173 183 221 222 223 224 225 243 249 271 273 286 338 382 383 398 399 438 539 552 558 559 585 586
rs16901941^0^	rs16786^1^	12 17 33 45 71 83 159 160 165 169 183 192 213 218 221 223 229 271 273 382 438 477 539 545 552 559 576
rs16901941^0^	rs2293775^1^	12 17 33 45 71 83 159 160 165 169 183 192 213 218 221 223 229 271 273 382 438 477539 545 552 559 576
rs16901941^0^	rs1805793^1^	12 17 33 45 71 83 159 160 165 169 183 192 213 218 221 223 229 271 273 382 438 477 539 545 552 559 576
rs16901941^0^	rs1805841^1^	12 17 33 45 71 83 159 160 165 169 183 192 213 218 221 223 229 271 273 382 438 477 539 545 552 559 576

Only the first 5 combinations are shown. All 43 combinations in Cluster 2 are shown in supporting S2 Table. ^0,1,2^ after rs number shows genotype (0 homozygous for the major allele, 1 heterozygous, 2 homozygous for the minor allele). Patient number 0-378 indicates oral cancer; patient number 379-631 indicates leuokoplakia.

The SNP genotypes in cluster 1 and cluster 2 and their genes are shown in Table 3. The SNP genotypes in cluster 1 belong to the double-strand break repair pathway, mismatch repair pathway, and DNA damage response pathway [14], whereas the SNP genotypes in cluster 2 belong to the double-strand break repair pathway only. Table 3 also shows that no overlap existed between the SNP genotypes in the combinations in the two clusters. Twelve patients were member of both clusters.

**Table 3:** The SNP genotypes that are found in the combinations in cluster 1 and cluster 2

genes	Cluster 1.	Cluster 2.
msh3	rs6151698^1^,rs3797893^1^, rs245383^1^, rs5008507^1^, rs3797896^1^, rs1650737^1^, rs245332^1^, rs245331^1^, rs26779^2^	
atr	rs11707731^0^	
atm	rs228589^1^	
nbn	rs13312928^1^, rs1805846^1^, rs7006322^1^, rs13312971^2^, rs2735384^1^	rs16901941^0^, rs2735388^1^, rs10090863^1^, rs7840099^1^, rs7829246^1^, rs16786^1^, rs741777^1^,rs6987873^1^, rs13278453^1^,rs2293775^1^,rs1805793^1^,rs1805794^1^, rs1805841^1^
xrcc5	rs207923^1^, rs207938^0^, rs207943^0^, rs9288516^1^, rs16855489^2^	rs828909^1^, rs828911^1^,rs705649^1^,rs828699^1^,rs828701^1^, rs828702^1^,rs207876^1^, rs207878^1^
mre11a	rs511184^1^, rs11820430^1^, rs13447719^1^, rs13447682^1^	rs521669^1^,rs584707^1^, rs552126^1^,rs3017077^1^, rs588701^1^,rs522596^1^,rs592068^1^,rs654718^1^
prkdc	rs8178107^1^, rs8178099^1^, rs8178176^1^, rs8178057^1^	rs6995756^1^, rs7003908^1^
rad50	rs6596084^2^, rs2706357^1^	rs2706338^1^,rs2244012^1^,rs2706347^1^,rs2246176^1^, rs12187537^1^,rs2522394^1^,rs2106984^1^,rs17772583^1^, rs2237060^1^, rs2040704^1^, rs2074369^1^
xrcc6		rs132774^2^

Mismatch repair pathway: msh3. DNA damage response pathway: atr, atm. double strand breaks repair pathway: nbn, xrcc5, mre11a, prkdc, rad50, xrcc6. ^0,1,2^ after rs number shows genotype (0 homozygous for the major allele, 1 heterozygous, 2 homozygous for the minor allele).

All of the SNP genotype combinations in the clusters were found exclusively in patients. As many of the patients contributed several combinations to the clusters, it was possible to map the combinations for the patients, creating a larger combination or pattern of SNP genotypes for each patient in the clusters. The personal patterns in cluster 1 contained from 2 to 16 SNP genotypes, whereas the patterns in cluster 2 contained from 7 to 35 SNP genotypes. A typical pattern for a patient in each cluster is shown in Table 4. All the patterns for the 191 patients in cluster 1 and the 54 patients in cluster 2 are shown in Supporting S4 and S5 Tables.

**Table 4:** Examples of the personal pattern of SNP genotypes for two patients

Patient in cluster 1	rs228589^1^ rs3797896^1^ rs8178176^1^ rs13447682^1^ rs2706357^1^ rs8178057^1^ rs9288516^1^
Patient in cluster 2	rs16901941^0^ rs132774^2^ rs16786^1^ rs2293775^1^ rs1805793^1^ rs1805841^1^ rs2244012^1^ rs2706347^1^ rs2246176^1^ rs2522394^1^ rs521669^1^ rs2106984^1^ rs2040704^1^ rs2706338^1^ rs12187537^1^ rs2074369^1^ rs1327702 rs2735388^1^

The personal patterns of SNP genotypes for the 191 patients in cluster 1 are shown in Supporting S4 Table. The personal patterns of SNP genotypes for the 54 patients in cluster 2 are shown in Supporting S5 Table. 0,1,2 after rs number shows genotype (0 homozygous for the major allele, 1 heterozygous, 2 homozygous for the minor allele).

Of the 373 patients with oral cancer, 55% were included in the clusters; in contrast, only 16% of the 253 patients with leukoplakia were included in the clusters. Supporting S4 and S5 Tables show that the personal patterns of SNP genotypes in the leukoplakia patients were similar to the patterns of the oral cancer patients in the respective clusters.

## DISCUSSION

In the present study of 325 SNPs from 11 genes related to DNA repair, 46,469 combinations of two SNP genotypes were found exclusively in patients with oral cancer or leukoplakia. Among these combinations two clusters of combinations were found to be significantly associated to oral cancer and leukoplakia. The clusters contained respectively 52 and 43 combinations, and 191 and 54 patients. It was not the single combination in the two clusters, but the clusters as such that were significantly associated to oral cancer and leukoplakia, as neither one nor two clusters of this size or larger were found in 1000 permutations. The two clusters were very different from each other because no overlap was seen between the SNPs in the combinations in the two clusters indicating the occurrence of two completely different genetic subgroups of patients with oral cancer (Table 3).

A significant cluster can be seen as a general risk factor for oral cancer, and accumulation in the genome of combinations belonging to the cluster can be seen as a personal risk factor for the single patient. Almost all of the patients had a personal pattern of combinations in their genome, indicating an extreme genetic heterogeneity. However, within a cluster these patterns were similar, hereby restoring a kind of genetic homogeneity. The number of SNP genotypes in the patterns in cluster 1 was much smaller (2-16), than the number of genotypes in the patterns in cluster 2 (7-35), (Supporting S4 and S5 Tables). Despite the low number of SNP genotypes in the patterns in cluster 1, these patterns contained genotypes from three different DNA repair pathways, whereas the many SNP genotypes in the patterns in cluster 2 were from only one pathway, suggesting that the accumulation of few genetic variants in several key pathways may result in the same risk of disease as the accumulation of many genetic variants in a single pathway.

The difference between the two genetic subgroups raises the question about the occurrence of corresponding clinical subgroups of patients. In a previous study of combinations of genetic data in patients with bipolar disorder, genetic subgroups were found to correspond to clinical subgroups [11, 15]. However, in the study [14] that served as the basis for the present study, the clinical data of interest were related to tobacco use and were not significantly associated to the clusters.

A goal of genetics is to contribute to diagnosis and prediction of disease. As 55 % of the patients with oral cancer were included in the clusters, the combinations in the clusters may be markers that can be used as a supplementary tool regarding diagnosis of this disease. Leukoplakia is a condition that may develop into oral cancer [17], emphasizing the importance of prediction for patients with this condition. Thus it was interesting to see if patients with leukoplakia were present in the clusters. Compared with the many patients with oral cancer in the clusters, only 16% of patients with leukoplakia were found in the clusters. A prospective study may show if oral cancer will occur more often in these 16%, than in the 84% of the leukoplakia patients outside the clusters. The presence of patients with leukoplakia in both clusters shows that also these patients can be divided into two genetic subgroups. Supporting S4 and S5 Tables show that the personal patterns of SNP genotypes in the leukoplakia patients were similar to the patterns of the oral cancer patients in the respective clusters, suggesting that mapping of such patterns may be a useful tool regarding prediction of risk for oral cancer in patients with leukoplakia.

It is noteworthy that combinations of as little as two SNP genotypes resulted in clusters of these combinations significantly associated to oral cancer or leukoplakia and containing more than half of the oral cancer patients. Similar results may be obtained from many genetic studies that have used the single locus strategy, if these studies are supplemented with an analysis of combinations of their already available data.

## MATERIALS AND METHODS

### SNP genotype data

Procedures for collection of blood samples and written informed consent form were reviewed and approved by the Institutional Ethical Committee, CSRI-Indian Institute of Chemical Biology, Kolkata, India. Written informed consent was obtained from all case and control subjects. Subjects (535 control persons, 373 patients with oral cancer, 253 patients with leukoplakia), genes, SNP selection, and genotyping have previously been described in detail [14].

### Combinations and statistics

The theoretical number of combinations of two SNPs from among 325 SNPs is 325!/(325-2)!2! = 52,650. The theoretical number of combinations of two SNP genotypes from among 325 SNP is 52,650 × 3^2^ = 473,850 because each SNP corresponds to three genotypes, and each combination of two SNPs corresponds to 3^2^ combinations of two SNP genotypes.

### A: Preparation of data

The SNP genotypes of control subjects and patients can be listed in a table with 1161 rows (373 for patients with oral cancer + 253 for patients with leukoplakia + 535 for controls) and 325 columns ( for the SNPs).

### B: Analysis of combinations of two SNP genotypes

Each of the 52,650 SNP pairs is analyzed one at a time. For each SNP pair the number of occurrences of the 9 SNP genotype combinations ((0 0)(0 1)(0 2)(1 0)(1 2)(1 2)(2 0)(2 1)(2 2)) are counted in the 1161 subjects (e.g., using the Excel function COUNTIFS). If a SNP genotype combination is found only in the group of patients, the combination is stored with the indices of the associated patients.

Repeating step 1 for the 52,650 SNP combinations by a loop function resulted in 46,469 combinations occurring exclusively in the patients. Of the 46,469 patient-specific combinations 25,179 occurred only once; 10,969 combinations were common for two patients; 4,250 combinations were common for three patients, and the highest number having a common combination was 57 patients, who shared one combination.

### C: Analysis of combinations significantly associated to oral cancer

Permutation tests showed that the 46,569 combinations found exclusively in patients could be a random finding. However, combinations common for many patients may more likely be significantly associated to oral cancer, compared with those that are common for few patients. But again permutation tests showed that also combinations common for many patients could be random findings. The probability of finding combinations significantly associated to oral cancer may also be increased by analysis of combinations sharing a SNP genotype that occurred more often in patients than in controls, compared with SNP genotypes distributed equally between patients and controls.

This led to the following strategy: Select combinations found exclusively in patients, among these select those that are common for many patients and among these select those containing SNP genotypes that occur more often in patients than in controls. Starting with the largest groups of patients that share combinations and SNP genotypes that have the highest occurrence among patients, this strategy allowed identification of clusters of combinations of two SNP genotypes that could be analyzed by permutation tests.

### D. Permutation test

Each cluster is analyzed by a permutation test, using 1000 permutations of the whole material of 535 control subjects and 626 patients. Only clusters with many patients and many combinations with a common SNP genotype obtained statistical significance. As soon as clusters became too small to obtain statistical significance no further clusters were analyzed, the limit was a p-value > 0.05, (when more than 50 clusters, of the same or larger size as the original cluster, were found in the 1000 permutations). The p-values for all significant clusters were corrected by Bonferroni correction.

Using the above steps in the present study, eight clusters were found significantly associated to oral cancer, seven of these clusters showed overlap with respect to SNP genotypes and patients and were merged into one cluster. Permutation tests of the two remaining clusters resulted in p-values < 0.001.

Figure 1 summarizes the steps leading from 325 SNPs to clusters of combinations of SNP genotypes significantly associated to oral cancer or leukoplakia.

## SUPPLEMENTARY TABLES


